# Tapentadol Prolonged Release Reduces the Severe Chronic Ischaemic Pain and Improves the Quality of Life in Patients with Type 2 Diabetes

**DOI:** 10.1155/2018/1081792

**Published:** 2018-02-20

**Authors:** Anna Tedeschi, Alessandra De Bellis, Piergiorgio Francia, Arianna Bernini, Marco Perini, Elisabetta Salutini, Roberto Anichini

**Affiliations:** ^1^Diabetes Unit and Diabetes Foot Unit, San Jacopo Hospital, Pistoia, Italy; ^2^Department of Clinical and Experimental Medicine, School of Human Health Sciences, Florence, Italy

## Abstract

This study has been performed in diabetic type 2 patients with pain due to peripheral artery disease (PAD) in order to evaluate the efficacy and tolerability of tapentadol prolonged release (PR). *Methods*. 25 patients with type 2 diabetes (13 F and 12 M) were admitted in the study. The evaluation of the analgesic efficacy of tapentadol PR was based on both the assessment of the intensity of the pain (NRS scale from 0 to 10) and the nature of the pain (DN4 questionnaire) and on assessment of the patient's quality of life and state of health (SF-12 Health Survey). Study duration was 3 months: a baseline visit and follow-up included visits after 1 week, 1 month, 2 months, and 3 months. *Results*. At the beginning of the study, the mean intensity of the pain was 7.88 ± 1.17 on the NRS scale and at visit 2 it reduced in a statistically significant way; at the end of the treatment with tapentadol PR, the mean intensity was 2.84 points on the NRS scale. *Conclusion*. In type 2 diabetic patients with chronic severe pain due to PAD, tapentadol PR reduced pain intensity, improving the quality of life.

## 1. Introduction

According to WHO definition, diabetes mellitus is a chronic disease that appears when a deficit in the secretion of insulin by the pancreatic cells or an insulin resistance occurs [1]. In 2035, the overall diagnoses of the different types of diabetes could reach 595 million cases, an impressive impact due to the transformation of lifestyles and feeding [2].

According to the International Diabetic Foot study group, a diabetic foot is defined as a foot with anatomical-functional alterations caused by peripheral artery disease (PAD) and/or distal symmetrical neuropathy.

Neuropathy and arteriopathy often appear in the same patient and are capable of generating not only a significant worsening of the patient's prognosis and quality of life but also chronic complex pain [3–5].

Diabetic neuropathy is frequent and affects up to 50% of patients with types 1 and 2 diabetes [3, 4]; it is a condition that is often severe and disabling and also characterised by the presence of neuropathic pain.

Diabetic PAD affects medium and small arteries (the distal part of the superficial femoral, popliteal, and subgenicular arteries), with relatively less involvement of the aorto-iliac arteries than in atherosclerotic arteriopathies [5].

Clinically, a pain presented during the march is less prevalent than in nondiabetic patients due to the possible simultaneous presence of polyneuropathy, so it is not rare that the first clinical manifestation of the disease is skin ulceration [5].

The pain that accompanies arteriopathy is often intense, with a complex pathogenesis, due to the presence of both a nociceptive and a neuropathic component, initially triggered by walking and then also present at rest, as the vascular damage progresses [6–9].

It follows that an appropriate analgesic treatment has to overcome the intensity and the nociceptive and neuropathic components of the pain. At the moment, this is possible by associating drugs with a different action, such as an analgesic and an antidepressant or gabapentinoid, or by using a single, dual-acting analgesic, such as tapentadol, which presents opioid action, mediated by *μ* receptor agonism (MOR), synergic with a noradrenergic action due to the inhibition of noradrenalin reuptake (NRI, noradrenaline reuptake inhibitor) [10–14].

The analgesic action of tapentadol has been studied in several animal models of neuropathic pain which demonstrated that it has a marked and specific activity, more complete than the action of traditional opioid such as morphine [12–16]. Tapentadol was also found to be more efficacious than gabapentine in neuropathic pain caused by compression or by the lesion of a peripheral nerve (in animal models of chronic constriction or spinal nerve ligation), diabetic polyneuropathy (in the streptozotocin-induced diabetes model), or chemotherapy (vincristine polyneuropathy model) [6–15].

A totally new piece of information emerged in a recent study of patients with diabetic polyneuropathy, in which tapentadol prolonged release (PR) (mean dose 433 mg/day) was able to increase descending pain modulation after 4 weeks of treatment [16]. We can therefore deduce that in patients with chronic pain due to diabetic neuropathy, the analgesic effect of tapentadol is a result of activation of descending noradrenergic inhibition.

Finally, the clinical data on the use of tapentadol PR in patients with polyneuropathic pain was derived from two double-blind randomised phase III studies against placebo [17–19]. Already in the titration phase, the mean pain intensity decreased in a way that was both clinically and statistically significant (over 3 points on a numerical scale from 0 to 10, *P* < 0.001). In the double-blind phase, this improvement was only maintained in those patients who were receiving tapentadol PR at a mean daily dose of 370–420 mg.

So based on these data in the literature, collected on patients with painful diabetic polyneuropathy, we decided to perform a study in diabetic patients with pain due to peripheral arteriopathy disease (PAD) in order to evaluate the efficacy and tolerability of tapentadol PR.

## 2. Materials and Methods

This is an observational cohort study. It has been performed following the Declaration of Helsinki ethical standards and has been approved by a Research Ethics Committee of USL Centro Tuscany with identification number 11977. The study was carried out at the Diabetes Foot Unit of San Jacopo Hospital of Pistoia, Tuscany, Italy, using an observational design to evaluate the analgesic efficacy and tolerability of tapentadol PR in type 2 diabetic patients with PAD.

25 adult patients of both sexes (13 F and 12 M) aged between 45 and 86 years (mean ± SD 65.5 ± 20.5) affected by type 2 diabetes, with chronic ischaemic pain, or mixed (ischaemic and neuropathic) pain were enrolled in the study (Table 1).

They were able to provide informed consent. Patients with chronic pain of a different nature or oncological pain and patients under 18 years of age and pregnant or breast-feeding women were not included in the study. Patients were enrolled in June 2015 and followed until February 2016.

The study lasted 3 months. Upon admission (baseline visit, V0), in addition to the primary pathology, the characteristics of the pain (duration, triggering factors, and trend over time), previous analgesic treatments, including details of their tolerability, and concomitant diseases and treatments were noted. Following visits were performed after 1 week (V1), 1 month (V2), 2 months (V3), and 3 months (V4).

**Figure 1:**
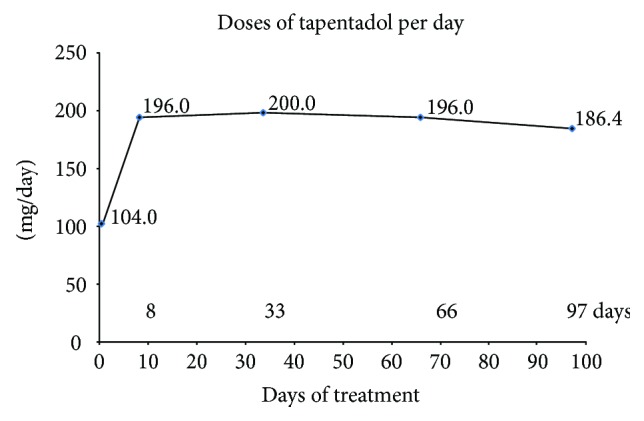
Mean daily dosages of tapentadol PR administered in 25 diabetic patients with chronic pain consequent on peripheral artery disease (PAD).

Tapentadol PR was administered according to the therapeutic indications, posology, and warnings for use stated in the Summary of Product Characteristics. Specifically, the daily dosage at the start of treatment was 100 mg/day in 24 patients, while 200 mg/day was prescribed in a single patient; at subsequent visits, the mean daily dosage was approximately 200 mg/day, with a minimum of 100 mg/day and a maximum of 300 mg/day (Figure 1).

The evaluation of the analgesic efficacy of tapentadol PR was based on the intensity of the pain reported at each visit (numeric pain intensity scale, NRS scale from 0 to 10) and the nature of the pain (diagnostic neuropathic pain, DN4 questionnaire), [20, 21], as well as the patient's quality of life and state of health (Short Form-12 Health Survey; SF-12 [21]).

Patients were considered responders if, after treatment with tapentadol PR, the intensity of pain recorded on the NRS scale fell by 30% or 50% from the baseline value.

The DN4 is a questionnaire developed by Bouhassira et al. that includes the use of both verbal descriptors and the neurological signs noted by examining the patient [20, 21]. Each positive response is scored with 1 point, and each negative response with 0. The cut-off for a diagnosis of neuropathic pain is a total score of 4/10; if the score is ≥4, there is a greater probability that the patient is suffering from neuropathic pain (sensitivity = 82.9%; specificity = 89.9%).

Sleep quality was also assessed, on a 4-point scale (deep, good, frequent awakening, and very disturbed), and the number of patient responders at 30% and at 50% was assessed. Specifically, those patients who had recorded a reduction in the score for pain intensity of at least 30% or 50% from the baseline level measured on the NRS were considered responders.

For the evaluation of tolerability, side effects were recorded during the appointments, noting their severity, duration, relationship with the ongoing treatment, and action taken.

The statistical analysis was carried out as follows [22–25]. The parameters measured at the baseline have been presented descriptively, and specifically, continuous values as means, standard deviation (SD), minima, and maxima, while the discrete values have been presented as frequencies with relative percentages.

The pain variable (numerical pain score) and the DN4 questionnaire variable were analysed with a nonparametric test, the Mann–Whitney *U* test.

Other frequency variables were analysed in contingency tables using the *χ*^2^ test.

The frequencies of responder patients at appointments 2, 3, and 4 were analysed using the binomial test with a 95% confidence interval, for both a 30% and a 50% reduction.

The mean daily pain and the DN4 questionnaire scores measured at the start and at the various control appointments during the treatment with tapentadol PR were analysed using Friedman's nonparametric test for nonindependent data.

Sleep quality was analysed against the baseline at each appointment, using the McNemar test.

The Health Survey, SF12, developed in accordance with Schwartz et al.'s paper [20], was analysed using the Friedman's nonparametric test (Table 2).

## 3. Results

### 3.1. Population

25 patients with type 2 diabetes have been studied: 22 cases presented distal ischaemic ulcerative lesions, 2 cases presented ischaemic leg lesions, and there was one case of ischaemic in the lower limbs without ulcers.

Of the entire cohort, 9 patients were being treated with insulin, 10 with metformin, 1 with insulin and metformin, 1 with GLP-1 agonist, and, finally, 2 patients were being treated by diet only.

The 3-month study was completed by all 25 subjects; treatment was then suspended in 2 cases due to complete regression of the pain, and in another case, due to a switch to morphine.

All patients attended 5 visits: at the start of the observation, V0 after 7 ± 2 days (mean ± SD), V1 after 33 ± 3 days, V2 65 ± 11 days, and V3 96 ± 11 days.

In terms of pain history, the pain was defined as prevalently neuropathic in 6 patients, mixed in 12 patients, and ischaemic in 7 patients. Four patients referred continous pain, while 21 intermittent. Triggering factors identified were movement in 15 patients, loading in 17 patients, while in 16 cases, the pain was spontaneous and also present at rest.

All patients were already being treated for pain relief (nonsteroidal anti-inflammatory drugs in 18 cases, opioids in 7 cases, gabapentinoids in 5, paracetamol in 3, and antidepressants in 3); these treatments had little effect in 20 patients, although generally well tolerated (21 cases). Stypsis was reported in 2 subjects.

Upon admission to the study, the mean intensity of the pain was 7.88 ± 1.17 (mean ± SD; min–max: 6–10) and sleep quality was compromised in all patients (very disturbed in 8 cases, frequent awakenings in 17 cases).

All patients were assessed with 2 questionnaires at the baseline: the DN4 for the assessment of neuropathic pain and the SF-12 Health Survey.

The result of the DN4 questionnaire confirmed the presence of neuropathic pain (score ≥ 4) in 18 of the 25 patients.

As for the SF-12 Health Survey, the physical health scores were between 19.4 and 38 (27.06 ± 5.28, mean ± SD) while the mental health scores were between 19.1 and 47 (28.77 ± 9.42, mean ± SD).

Finally, 16 patients presented concomitant conditions for which they were receiving specific treatment: arterial hypertension in 11 cases, cardiopathy in 3 cases, chronic obstructive pneumopathy disease in one case, and hypercholesterolaemia in one case.

### 3.2. Analgesic Efficacy

**Figure 2:**
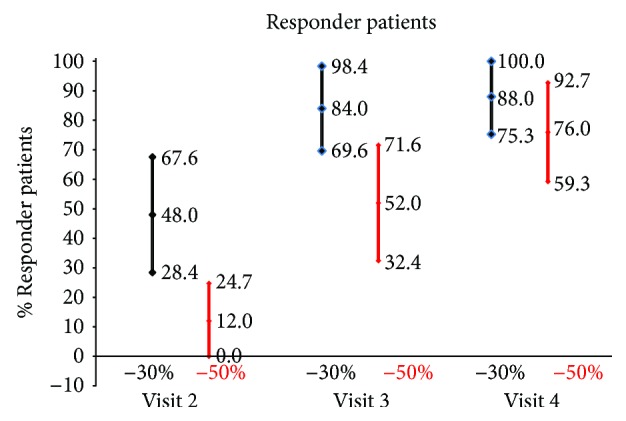
Frequency of responder subjects at 30% and 50% of 25 diabetic patients with chronic pain consequent on peripheral arteriopathy disease with tapentadol PR for 3 months.

The frequencies of responder subjects at both 30% and 50% were high and progressive during the study (Figure 2).

The frequency of 30% responder subjects was 48.0% at the first visit (12/25 patients, confidence interval, CI 95%: 28.4% ÷ 67.6%); 84.0% (21/25 patients, CI 95%: 69.6% ÷ 98.4%) at visit 2, 88.0% (22/25 patients, CI 95%: 75.3% ÷ 100.0%) at visit 3, and 92.0% (23/25 patients; CI 95%: 82.4% ÷ 100.0%) at visit 4.

The frequency of 50% responder subjects was 12.0% (3/25 patients, confidence interval, CI, 95%: 0.0% ÷ 24.7%) at the first visit, 52.0% (13/25 patients; CI 95%: 32.4% ÷ 71.6%) at visit 2, 76.0% (19/25 patients; CI 95%: 59.3% ÷ 92.7%) at visit 3, and 80.0% (20/25 patients; CI 95%: 61.4% ÷ 94.3%) at visit 4.

The intensity of the pain was significantly reduced during the study, being already statistically significant at visit 2 (*P* < 0.01); at the end of the period of treatment with tapentadol PR, the mean intensity was 2.84 points on the NRS scale (5 points below the baseline, *P* < 0.01).

As for questionnaire DN4, the frequency of patients with neuropathic pain decreased rapidly and significantly, from 18 (72%) to 9 patients (36%) at visit 2, to 3 patients (12%) at visit 3, and to 4 patients (16%) at visit 4 (*P* < 0.001).

Sleep quality, initially poor in all subjects, improved during the period of treatment, compared to the baseline, in a statistically significant way (*P* < 0.01); at the end of the study, only 4 subjects still reported frequent awakenings (1 case) or very disturbed sleep (3 cases).

As for Health Survey SF-12, both the physical status score and the mental status score increased during the treatment period in a statistically significant way (*P* < 0.01).

### 3.3. Tolerability

Tolerability was good; no adverse events referable to the treatment with tapentadol PR were reported.

The incidence of stypsis during the period of treatment with tapentadol PR did not increase in a statistically significant way; specifically, in addition to the 2 subjects (8%) with stypsis at the baseline, only 3 more patients (12%) reported the symptom at visit 1 and at visit 2.

## 4. Discussion

The results of our study demonstrate the good analgesic efficacy of tapentadol PR in type 2 diabetic patients with chronic pain consequent on PAD and/or ischaemic ulcerative lesions as well as neuropathic pain.

At the end of the study, the frequencies of responders at both 30% (88%) and 50% (76%) were very high, with a reduction in pain intensity of about 5 points on a numerical scale from 0 to 10, which was highly significant both clinically and statistically. In parallel with the pain control, night rest was also recovered and there was a net improvement in the quality of life.

These interesting results were obtained with tapentadol PR, at average doses of 200 mg/day, in a group of patients which, although numerically limited, presented pain which was complex for its notable intensity, for the frequent presence of a neuropathic component and for poor responsiveness to other treatments. In fact, all the patients had already been treated with various drugs for analgesia (nonsteroid antinflammatory drugs, opioids, gabapentinoids, paracetamol, and antidepressants) with little benefit, in most cases.

The prompt and significant pain relief response observed with tapentadol PR may depend, primarily, on its dual action mechanism, both opioid and noradrenergic (MOR-NRI [12–16]) that is able to reduce the pain itself and to modulate and strengthen the inhibitory control systems that affect pain transmission [12–16].

This is the first published information collected on a group of patients with a severe complication of diabetes mellitus such as PAD; in fact, to date, the analgesic efficacy of tapentadol PR has been demonstrated in diabetic polyneuropathic pain [19]; in particular, mean doses ranging from 100 to 200 mg/day for 15 weeks permitted a marked reduction in the intensity of the neuropathic pain (more than 3 points on the 0–10 NRS scale), so that tapentadol obtained specific recognition from the US FDA for this type of pain.

However, it is noteworthy that the sample examined is limited and it will be important to confirm these preliminary data in a greater number of diabetic patients. Moreover, the duration of treatment is quite short and can certainly be prolonged in order to verify the efficacy of the treatment in a longer period of time. The analgesic role of tapentadol PR was evaluated in patients treated with other drugs for other pathologies, and, as an observational study, we did not modify the concomitant therapy. The variable of concurrent therapies should therefore be considered in the interpretation of the results.

## 5. Conclusion

These preliminary data seem to demonstrate the efficacy of tapentadol PR in the treatment of ischaemic pain and not just of neuropathic pain. However, this result needs to be confirmed in a larger sample of diabetic patients who would need to be observed for a longer period of time.

Moreover, in our patients, tapentadol PR seems to be well tolerated and no adverse events have been reported, thus making this drug particularly interesting in the treatment not only of neuropathic pain but also of ischaemic pain.

## Figures and Tables

**Table 1:** Main and baseline characteristics in patients with diabetes and PAD.

Group	Patients	Males	Females	Males versus females *P* value
Gender (M/F)^∗^	12/13	12/0	0/13	—
Age (yrs)^§^ mean ± SD	74.4 ± 9.55	76.7 ± 5.8	72.2 ± 11.9	NS
Types of pain (%)^∗^				NS
Nociceptive pain	24.0	8.3	38.5	
Neuropathic pain	0	0	0	
Mixed	48.0	50.0	46.2	
Other	28.0	41.7	15.4	
Trends of pain (%)^∗^				NS
Continuous	16	16.7	15.4	
Intermittent	84.0	83.3	84.6	
Pain intensity (NRS)°	7.9	8.0	7.8	NS
DN4 score < 4 (%)^∗^	28.0	25	30.8	NS
Quality of sleep (%)				NS
Very disturbed	32.0	41.7	23.1	
Frequent awakenings	68.0	58.3	76.9	
Constipation (%)^∗^	8.0	16.7	0	NS
Side effects (%)^∗^	8.0	0	15.4	NS

Values are mean ± SD. Quality of sleep has been assessed on four-point scale: deep, good, frequent awakenings, and very disturbed. NRS: numeric pain intensity scale from 0 to 10. DN4: diagnostic neuropathic pain questionnaire. °Related to NRS, numeric pain intensity scale. ^§^By one-way ANOVA (*P* value) test. ^∗^By Chi-square method. By Mann–Whitney *U* test.

**Table 2:** Doses per day of tapentadol PR, pain intensity, quality of sleep, and quality of life at different time intervals between examinations in patients with diabetes and PVD.

	Baseline	V2	V3	V4	
Time intervals	0	33.3 ± 8.8	65.6 ± 11.1	96.8 ± 11.0	
Tapentadol PR (mg)	104.0 ± 20.0	200.0 ± 28.9	196.0 ± 35.1	186.4 ± 56.0	
NRS^∗^	7.9 ± 1.2	5.7 ± 1.9	3.9 ± 2.1	2.8 ± 2.3	<0.01
NRS responders (*N* = 25)					
Number of patients ≥ 30%	—	12	21	22	
Number of patients ≥ 50%	—	3	13	19	
Quality of sleep° (%)					<0.01^§^
Very disturbed	32	20	16	12	
Frequent awakenings	68	36	0	4	
Good	0	44	76	28	
Deep	0	0	8	56	
Presence of constipation° (%)	8	20	12	12	NS
DN4^∗^	4.0 ± 1.2	2.9 ± 1.3	1.8 ± 1.3	1.2 ± 1.5	<0.01
SF12^∗^					
PCS-12	27.1 ± 5.3	29.5 ± 6.1	43.8 ± 11.2	49.1 ± 10.8	<0.01
MCS-12	28.8 ± 9.4	36.2 ± 10.2	43.8 ± 11.2	49.1 ± 10.8	<0.01
Adverse events	0	0	0	0	

Values are mean ± SD. NRS: numeric pain intensity scale from 0 to 10. Responders: patients who had recorded a reduction in the NRS score of at least 30% and 50% from the baseline level measured. DN4: diagnostic neuropathic pain questionnaire. Quality of sleep was assessed on four-point scale: deep, good, frequent awakenings, and very disturbed. SF-12 summary scores (Physical Component Summary: PCS-12 and Mental Component Summary: MCS-12) also range from 0 to 100. ^∗^Friedman's test. °Test McNemar. ^§^Sleep quality and constipation have been assessed comparing each medical examination to baseline values.
